# Exploring the role of aging in the relationship between obstructive sleep apnea syndrome and osteoarthritis: Insights from NHANES data

**DOI:** 10.3389/fmed.2024.1486807

**Published:** 2024-11-28

**Authors:** Xin Luo, Minghong Chen, Jinghong Xu

**Affiliations:** ^1^Department of Geriatrics, Xiangya Hospital, Central South University, Changsha, Hunan, China; ^2^Department of Trauma Orthopedics, The First Affiliated Hospital of USTC, Division of Life Sciences and Medicine, University of Science and Technology of China, Hefei, Anhui, China

**Keywords:** aging, NHANES, osteoarthritis, obstructive sleep apnea syndrome, mediation analysis

## Abstract

**Background:**

Osteoarthritis (OA) is characterized by high morbidity and disability. While studies have demonstrated that OA is correlated with age-related diseases, few have shown the potential relationship between OA and obstructive sleep apnea syndrome (OSAS). OSAS is characterized by intermittent hypoxia and hypercapnia. We hypothesize that these stressors induce aging and increase the prevalence of OA.

**Methods:**

The study included 10,641 participants drawn from the National Health and Nutrition Examination Survey (NHANES) dataset during 2005–2008 and 2015–2018. The correlation between OSAS and OA was analyzed using multivariable logistic regression, aging-related biomarkers were calculated, and the role of aging was explored through mediation analysis.

**Results:**

OSAS was associated with an elevated risk of OA (for quartile 4 vs. quartile 1, odds ratio (OR) 2.31, 95% confidence interval (CI) 1.34 to 3.99; *p*-value for the trend = 0.004) after adjusting covariates. In the 20–59 years and > 60 years subgroup, the OSAS patients showed a similar trend (for quartile 4 vs. quartile 1, OR 5.69, 95% CI 2.75 to 11.8; *p*-value for the trend <0.001; OR 2.42, 95% CI 1.23 to 4.76; *p*-value for the trend = 0.004, respectively). Further mediation analysis revealed that aging acted as a mediator between OA and OSAS. The mediation proportions for biological age (BA) and phenotypic age (PA) were 13.82 and 52.94%, respectively, both with *p* < 0.001.

**Conclusion:**

These findings suggest that individuals with OSAS may have an increased prevalence of OA, with aging also being involved in the association.

## 1 Introduction

Osteoarthritis (OA) is a degenerative disease caused by the erosion of joint cartilage, leading to pain and disability. Estimates suggest that 250 million people have been affected by this condition (1). Previous studies have shown that elderly individuals diagnosed with arthritis are at a higher risk of developing chronic comorbidities, such as cardiovascular diseases (CVDs) and hypertension (2–4).

Obstructive sleep apnea syndrome (OSAS), primarily attributed to obesity and airway collapse, triggers a cascade of pathophysiological processes, including intermittent hypoxia and hypercapnia. These processes subsequently lead to hypertension, metabolic disorders, and inflammation, all of which share many similarities with chronic diseases (5). Existing studies have demonstrated that aging is associated with a decline in sleep quality. It has been frequently observed that sleep-disordered breathing and cognitive changes occur with age (6–8).

The incidence of OA and OSAS increases with age (7, 9). As people age, there is a growing trend toward the coexistence of multiple diseases, making the treatment process more challenging (10, 11). Although many studies have reported that OA is associated with chronic disease, few have shown the association between OA and OSAS in a large number of participants. The role of aging in these two diseases is still unknown.

Therefore, we used the National Health and Nutrition Examination Survey (NHANES, 2005–2008 and 2015–2018) database to explore the risk of OA and OSAS and the role of aging in this process. By managing OSAS and delaying aging, pain can be relieved and quality of life can be improved for elderly patients with OA.

## 2 Methods

### 2.1 Data source

**Figure 1:**
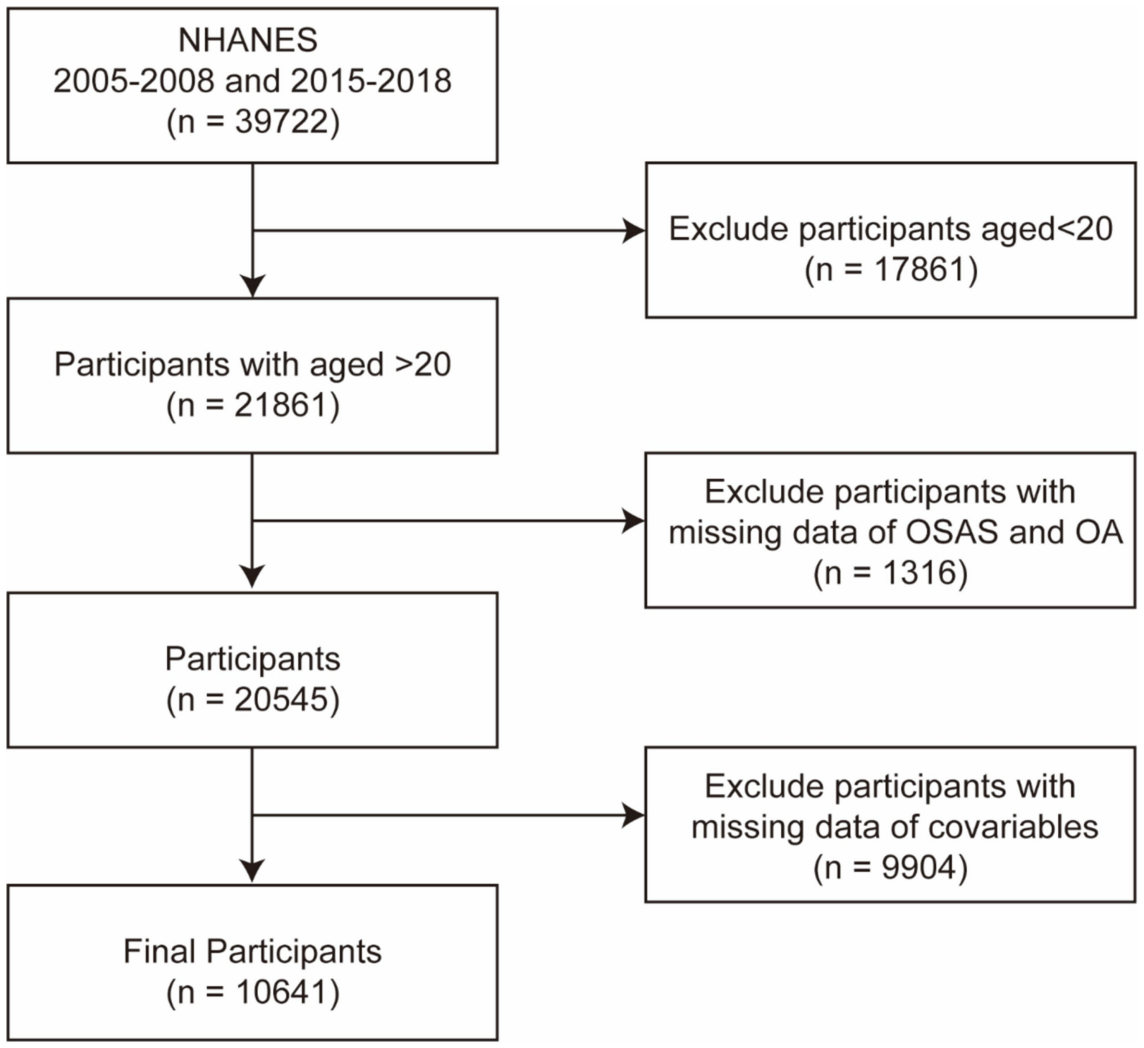
Flow chart of the participants in this study. NHANES, National Health and Nutrition Examination Survey; OA, osteoarthritis; and OSA, obstructive sleep apnea syndrome.

The NHANES is a database managed by the National Center for Health Statistics, created through representative sampling and supplemented with questionnaires, physical examinations, laboratory tests, and data collected from mobile examination centers. The Information Collection Review Office approved the research protocol involving human participants. We selected 2005–2008 and 2015–2018 as the study cycles because they included both OA and OSAS questionnaire data. From these cycles, we screened 21,861 individuals older than 20 years for this study (Figure 1), and 10,641 participants were included in the final analysis, with covariables incorporated. All participants provided written informed consent, and all collected information was maintained with strictly confidentiality.

### 2.2 Assessment of osteoarthritis

Studies have shown that 81% of osteoarthritis data collected through questionnaires are consistent with clinical diagnoses (12). Therefore, we used questionnaires from the NHANES dataset to collect relevant data. Individuals with self-reported osteoarthritis were included in the OA group, whereas those who were healthy, had other types of arthritis, and did not specify the type were included in the non-OA group.

### 2.3 Diagnosis of OSAS and the multivariable apnea prediction index

According to a previous study by Maislin et al., we used the OSAS questionnaire to calculate the multivariable apnea prediction (MAP) index and OSAS multivariable apnea prediction index value × 10 (OSAS.MAP10) based on information provided about snoring, sleep apnea, and fatigue severity (13–15) (see Supplementary material for details).

### 2.4 Measurement of the biological aging markers

Previous studies have shown that KDM biological age (BA) and phenotypic age (PA) more accurately predict an individual’s aging level through algorithmic assessments (16). We obtained data on individual BA biomarkers (C-reactive protein [CRP], serum creatinine, glycosylated hemoglobin, serum albumin, serum total cholesterol, serum urea nitrogen, serum alkaline phosphatase, and systolic blood pressure) and chronological age. We utilized the NHANES III data to compute BA and subsequently incorporated our data from the 2005–2008 and 2015–2018 cycles into the fitting process (17). Similarly, we obtained data on individual PA biomarkers (albumin, creatinine, glucose, C-reactive protein, lymphocyte percentage, mean cell volume, red blood cell distribution width, alkaline phosphatase, and white blood cell count) and used the BioAge R package to calculate phenotypic age (see Supplementary materials for details).

### 2.5 Potential confounders/other variables

We collected demographic and lifestyle data, including age (18), sex, ethnicity, educational background, family income ratio (PIR), smoking habits, and alcohol consumption, through detailed questionnaires. BMI and blood pressure data (the average of three blood pressure measurements, including those taken while using blood pressure-lowering drugs) were obtained from physical examinations and the medical conditions component of the questionnaires. By synthesizing self-reported health histories, medication records, and chronological age information, we evaluated the presence of cardiovascular diseases. This encompassed conditions such as coronary heart disease, congestive heart failure, myocardial infarctions, cerebrovascular accidents (strokes), and angina pectoris, as well as other chronic diseases including diabetes, asthma, and chronic obstructive pulmonary disease (COPD) (15).

### 2.6 Statistical analysis

This study used RStudio (version 4.4.0) for the statistical analysis of the weighted data. We grouped the participants according to their osteoarthritis status, and the Wilcoxon rank-sum test and the chi-squared test were used for demographic analysis. Quartiles were utilized to transform the continuous variable OSAS.MAP10 into a categorical variable. Multivariable logistic regression analysis was conducted to estimate the odds ratio (OR) and its 95% confidence interval (CI) for the association between OSAS.MAP10 and OA risk, adjusting for age, race, education level, PIR, BMI, smoking status, alcohol consumption, CVDs (coronary heart disease, stroke, heart attack, chronic heart failure, and angina pectoris), hypertension, diabetes, and COPD. Subsequently, the mediating role of aging in the relationship between OSAS.MAP10 and OA was investigated using the mediation package in RStudio. The quasi-Bayesian Monte Carlo method was employed, involving 1,000 simulations based on the normal approximation, to estimate the indirect effect (ACME, average causal mediation effect) and direct effect (ADE, average direct effect). The proportion of mediation was calculated by dividing the ACME by the total effect (ACME + ADE) (18).

## 3 Results

### 3.1 Basic clinical characteristics of the study participants

Table 1 shows the demographic characteristics according to the OA status. In this study, we screened 1,027 osteoarthritis patients aged 20 years or older. The OA group was older (60.47 ± 12.65) than the non-OA group (44.17 ± 15.29; *p* < 0.001). The OSAS.MAP10 score was higher in the OA group (4.82 ± 2.54; *p* < 0.001) compared to the non-OA group (OR 3.73 ± 2.53; *p* < 0.001). The participants who were female, had a high income, were obese, smoked, and consumed alcohol were more likely to have osteoarthritis (all *p*-values for the trend <0.001). The participants with a history of CVDs, hypertension, diabetes, or COPD also had a higher prevalence of osteoarthritis (all *p*-values for the trend <0.001). However, the participants with asthma did not show a significant difference in the prevalence of osteoarthritis (*p*-value for the trend = 0.2). Therefore, CVDs, hypertension, diabetes, and COPD were included in model 3.

**Table 1:** Baseline characteristics of the participants with and without OA.

Characteristic	Non-OA, *N* = 9,614 (89%)^1^	OA, *N* = 1,027 (11%)^1^	*p*-value^2^
Age, mean (SD), years	44.17 (15.29)	60.47 (12.65)	<0.001
Sex			<0.001
Male	5,206.00 (52.64)	406.00 (37.01)	
Female	4,408.00 (47.36)	621.00 (62.99)	
Race			<0.001
Mexican American	1,676.00 (8.63)	80.00 (2.35)	
Other Hispanic	850.00 (4.99)	56.00 (1.92)	
Non-Hispanic White	4,262.00 (70.25)	663.00 (84.78)	
Non-Hispanic Black	1,897.00 (9.49)	147.00 (5.35)	
Other/multiracial	929.00 (6.64)	81.00 (5.59)	
Education			0.045
Less than 9th grade	696.00 (3.27)	44.00 (1.93)	
9–11th grade	1,186.00 (8.48)	97.00 (6.85)	
High school graduate/GED	2,210.00 (22.98)	226.00 (22.06)	
Some college or AA degree	3,066.00 (32.83)	341.00 (32.43)	
College graduate or above	2,456.00 (32.43)	319.00 (36.74)	
PIR			<0.001
Low income	2,357.00 (15.98)	215.00 (13.22)	
Medium income	3,796.00 (35.02)	362.00 (29.77)	
High income	3,461.00 (49.00)	450.00 (57.01)	
Smoke			<0.001
Never	4,851.00 (51.92)	451.00 (45.56)	
Current	2,389.00 (23.29)	173.00 (15.34)	
Former	2,369.00 (24.80)	402.00 (39.09)	
Drinking	1,498.00 (14.29)	70.00 (5.68)	<0.001
BMI			<0.001
Normal	2,654.00 (29.65)	212.00 (19.90)	
Underweight	139.00 (1.36)	5.00 (0.52)	
Overweight	3,243.00 (32.44)	328.00 (32.04)	
Obese	3,578.00 (36.55)	482.00 (47.55)	
CVDs	743.00 (5.45)	192.00 (16.05)	<0.001
Hypertension	2,992.00 (27.32)	540.00 (47.64)	<0.001
Diabetes	906.00 (6.98)	157.00 (12.69)	<0.001
COPD	353.00 (3.21)	103.00 (9.04)	<0.001
Asthma	1,354.00 (14.49)	190.00 (16.53)	0.2
Arthritis	1,652.00 (15.49)	1,027.00 (100.00)	<0.001
OSA	4,907.00 (49.33)	613.00 (58.48)	<0.001
OSAS.MAP10	3.73 (2.53)	4.82 (2.54)	<0.001

1 median (IQR) for continuous variables; *n* (%) for categorical variables.

2 The Wilcoxon rank-sum test for complex survey samples.

The chi-squared test with Rao & Scott’s second-order correction. The continuous variables are presented as median. The categorical variables are presented as *n* (%). OA, osteoarthritis; OSA, obstructive sleep apnea syndrome; PIR, the ratio of family income to poverty; BMI, body mass index; CVD, cardiovascular disease; COPD, chronic obstructive pulmonary disease; OSAS.MAP10, OSAS multivariable apnea prediction index value × 10; SE standard error, *n*, number of participants; and %, weighted percentage. Median (SD) or median (IQR) for the continuous variables; *n* (%) for the categorical variables.

### 3.2 Associations between OSAS and OA risk

As shown in Table 2, the association between OSAS and OA was investigated using a multivariable logistic regression model, adjusting for potential covariates. In the unadjusted statistical model (model 1), quartile 4 of the OSAS.MAP10 scores was associated with an increased incidence of OA (OR 3.48, 95% CI 2.60 to 4.65, all *p*-values for the trend <0.005) compared to quartile 1. The observed trend was consistent with that of model 2 (OR 2.36, 95% CI 1.37 to 4.08, all *p*-values for the trend = 0.003, quartile 4 vs. quartile 1), in which we adjusted for age, sex, race, education level, PIR, BMI, smoking status, and alcohol consumption. Model 3 (in which the variables in model 2 and CVDs, hypertension, diabetes, and COPD were adjusted for) showed a similar trend (OR 2.31, 95% CI 1.34 to 3.99, all *p*-values for the trend = 0.004, quartile 4 vs. quartile 1).

**Table 2:** The association between the OSAS.MAP10 quartiles and OA.

	Model 1		Model 2		Model 3	
Characteristic	OR^1^ (95% CI^1^)	*p*-value	OR^1^ (95% CI^1^)	*p*-value	OR^1^ (95% CI^1^)	*p*-value
OSAS.MAP10 (continuous)	1.18 (1.14, 1.22)	<0.001	1.17 (1.09, 1.26)	<0.001	1.17 (1.09, 1.26)	<0.001
OSAS.MAP10 quartile						
Q1	Reference		Reference		Reference	
Q2	1.94 (1.46, 2.59)	<0.001	1.31 (0.92, 1.86)	0.129	1.30 (0.91, 1.85)	0.142
Q3	2.16 (1.60, 2.93)	<0.001	1.46 (0.98, 2.18)	0.063	1.47 (0.99, 2.18)	0.057
Q4	3.48 (2.60, 4.65)	<0.001	2.36 (1.37, 4.08)	0.003	2.31 (1.34, 3.99)	0.004
*p*-value for the trend	<0.001		0.003		0.004	

1 OR, Odds Ratio; CI, 95% CI.

Model 1: no covariates were adjusted. Model 2: adjusted for age, sex, race, education level, PIR, BMI, smoking status, and alcohol consumption. Model 3: adjusted for the covariates in model 2 and CVDs (coronary heart disease, stroke, heart attack, chronic heart failure, and angina pectoris), hypertension, diabetes, and COPD. OSAS, obstructive sleep apnea syndrome and OSAS.MAP10, OSAS multivariable apnea prediction index value × 10.

### 3.3 The associations between OSAS and OA risk in age and sex subgroups

Table 3 shows the associations between OSAS and OA risk in different age and sex subgroups. We found that the highest quartile of OSAS.MAP10 (OR 3.08, 95% CI 2.04 to 4.64, *p* < 0.001) in the 20–59 years subgroup was associated with an increased risk of OA after adjustment in model 1 compared to quartile 1. Model 2 (OR 8.18, 95% CI 4.24 to 15.8, *p* < 0.001) and model 3 (OR 5.69, 95% CI 2.75 to 11.8, *p* < 0.001) showed similar trends. However, OSAS.MAP10 in the >60 years subgroup did not show the trend (OR 1.01, 95% CI 0.58 to 1.77, *p* = 0.664) in model 1. After adjusting for the covariates, OSAS.MAP10 restored the trend in model 2 (OR 2.29, 95% CI 1.19 to 4.38, *p* = 0.006) and model 3 (OR 2.42, 95% CI 1.23 to 4.76, *p* = 0.004).

**Table 3:** The association between OSAS.MAP10 and OA in the age and sex subgroup.

		Model 1		Model 2		Model 3	
Group	Characteristic	OR^1^ (95% CI^1^)	*p*-value	OR^1^ (95% CI^1^)	*p*-value	OR^1^ (95% CI^1^)	*p*-value
20–59 years	OSAS.MAP10 quartile		<0.001		<0.001		<0.001
	Q1	Reference		Reference		Reference	
	Q2	1.52 (0.99, 2.34)		2.22 (1.37, 3.59)		2.01 (1.24, 3.25)	
	Q3	1.81 (1.20, 2.72)		3.56 (2.19, 5.80)		2.92 (1.78, 4.79)	
	Q4	3.08 (2.04, 4.64)		8.18 (4.24, 15.8)		5.69 (2.75, 11.8)	
	*p*-value for the trend	<0.001		<0.001		<0.001	
60+ years	OSAS.MAP10 quartile		0.664		0.006		0.004
	Q1	Reference		Reference		Reference	
	Q2	1.18 (0.66, 2.11)		1.24 (0.70, 2.19)		1.27 (0.71, 2.27)	
	Q3	0.97 (0.58, 1.61)		1.36 (0.78, 2.38)		1.44 (0.81, 2.55)	
	Q4	1.01 (0.58, 1.77)		2.29 (1.19, 4.38)		2.42 (1.23, 4.76)	
	*p*-value for the trend	0.664		0.006		0.004	
Male	OSAS.MAP10 quartile			<0.001			0.022
	Q1	Reference		Reference		Reference	
	Q2	2.25 (0.70, 7.22)		1.11 (0.34, 3.59)		1.09 (0.34, 3.52)	
	Q3	4.47 (1.71, 11.7)		1.24 (0.46, 3.36)		1.20 (0.44, 3.26)	
	Q4	13.7 (5.33, 35.1)		2.37 (0.82, 6.83)		2.27 (0.79, 6.55)	
	*p*-value for the trend	<0.001		0.022		0.028	
Female	OSAS.MAP10 quartile			<0.001			0.043
	Q1	Reference		Reference		Reference	
	Q2	2.70 (1.98, 3.69)		1.29 (0.85, 1.97)		1.29 (0.84, 1.97)	
	Q3	3.94 (2.76, 5.63)		1.60 (0.90, 2.85)		1.60 (0.91, 2.83)	
	Q4	6.24 (4.26, 9.14)		2.32 (1.25, 4.31)		2.16 (1.14, 4.07)	
	*p*-value for the trend	<0.001		0.043		0.096	

1 OR, Odds Ratio; CI, 95% CI.

Model 1: no covariates were adjusted. Model 2: adjusted for age, sex, race, education level, PIR, BMI, smoking status, and alcohol consumption. Model 3: adjusted for the covariates in model 2 and alcohol consumption, CVDs (coronary heart disease, stroke, heart attack, chronic heart failure, and angina pectoris), hypertension, diabetes, and COPD. OSAS, obstructive sleep apnea syndrome and OSAS.MAP10, OSAS multivariable apnea prediction index value × 10.

Table 3 shows that the highest quartile of OSAS.MAP10 in the male subgroup was associated with an increased risk of OA in all three models (all *p*-values for the trend <0.005). However, quartile 4 of OSAS.MAP10 in the female subgroup was associated with an increased risk of OA in model 1 (OR 6.24, 95% CI 4.26 to 9.14, *p* < 0.001) and model 2 (OR 2.32, 95% CI 1.25 to 4.31, *p* = 0.043), but there was no significant difference in model 3 (OR 2.16, 95% CI 1.14 to 4.07, *p* = 0.096).

### 3.4 Aging-mediated effects on the association between OSAS and OA risk

**Figure 2:**
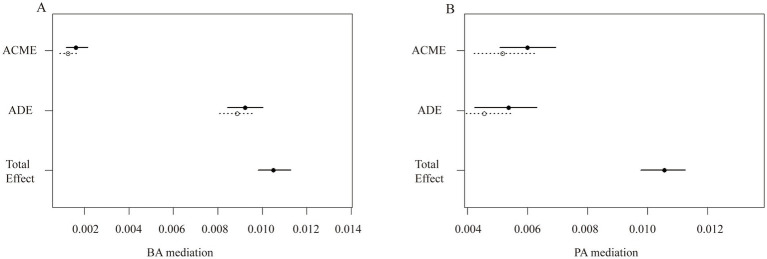
The aging-mediated plot illustrates the effects of aging on the association between OSAS with OA risk. (A) BA mediated the association between OSAS and OA. (B) PA mediated the association between OSAS and OA. BA, biological age; PA, phenotypic age; ACME, average causal mediation effect; and ADE, average direct effect; Total effect, ACME + ADE.

Furthermore, the mediating role of aging in the relationship between OSAS.MAP10 and OA is shown in Figure 2. In the phenotypic age group, the ACME was 0.00559 (95% CI: 0.00464, 0.01), the ADE was 0.00497 (95% CI: 0.00390, 0.01), and the proportion of mediation was 52.9%. In the biological age group, the ACME was 0.00145 (95% CI: 0.00104, 0.00), the ADE was 0.00905 (95% CI: 0.00824, 0.01), and the proportion of mediation was 13.8% (all *p* < 0.001).

## 4 Discussion

In this study, we identified two novel findings. First, OSAS was positively associated with OA in this cross-sectional investigation, which included 10,641 participants. Second, after adjusting for the covariates, aging was found to play a mediating role between OSAS and OA.

OSAS is a sleep disorder characterized by intermittent hypoxia and fragmented sleep, leading to oxidative stress and systemic inflammation. This condition is not only associated with aging-related diseases but may also accelerate the aging process (19, 20) and contribute to the hallmarks of aging, such as telomere attrition, mitochondrial dysfunction, and intercellular communication (21–24). Studies have found that OA shares the above risk factors (25–27). Silva et al. found that elderly patients with OA in the knees with OSAS are more prone to pain, stiffness, and physical dysfunction compared with those without OSAS (28). Carroll et al.’s investigation uncovered a connection between OSAS and shortened leukocyte telomere length, suggesting an association with accelerated biological aging (29). Vicente etal. found a reduction in certain biomarkers of inflammation in pharyngeal lavage samples collected from moderate-to-severe OSAS patients, who were treated with continuous positive airway pressure (30). In our study, compared to the control group, the participants diagnosed with OSAS exhibited higher levels of aging-related clinical biomarkers, including CRP and immunity-related cells.

Aging-induced alterations in articular cartilage particularly contribute to the progression of OA. Loeser et al. reported several mechanisms associated with aging that contribute to OA. Among these are inflammaging, cellular senescence, mitochondria dysfunction, and heightened oxidative stress (31). A recent study discovered that senolytic therapies, which specifically remove senescent cells, effectively decreased oxidation-modified proteins in aged arthritic knee joints, reduced pain, and promoted cartilage development (32, 33). It suggested that aging plays a critical role in OSAS and OA by promoting inflammation and oxidative stress. Our study is consistent with the findings of the studies mentioned above.

We used the NHANES database to explore the mediating effect of aging on OSAS and OA. Based on these findings, efficiently managing OSAS might serve as a strategy for delaying aging in clinical practice and alleviating patients’ pain. This approach offers new perspectives on minimizing the incidence of OA and enhancing the overall well-being of elderly patients.

Our study has certain strengths. We explored the association between OSAS and OA using a large population database. Furthermore, we evaluated the mediating effect of biological aging on the relationship between OSAS and OA, providing a more nuanced understanding of the relationship. This study includes some limitations. First, while we found a positive correlation between OSAS and OA, the observational study design precluded establishing causality. Future research should include longitudinal data collection and prospective studies to further explore this connection. Second, the data on OA and OSAS were self-reported, which introduced recall bias and decreased the credibility of the results. Third, although we adjusted for the survey cycles, the data on self-reported OA and OSAS were only available in four specific cycles. Therefore, future studies are needed to verify, evaluate, and reinforce our findings.

## 5 Conclusion

Our results suggest that OSAS may increase the prevalence of OA. In addition, we explored the mediating effects of aging on these two diseases. Our study could provide new insights into the management of various health concerns in the elderly population.

## Data Availability

The datasets presented in this study can be found in online repositories. The names of the repository/repositories and accession number(s) can be found in the article/Supplementary material.

## References

[1] Hunter DJ Bierma-Zeinstra S. Osteoarthritis. Lancet. (2019) 393:1745–59. doi: 10.1016/S0140-6736(19)30417-9, PMID: 31034380

[2] Xiao Q Cai B Yin A Huo H Lan K Zhou G. L-shaped association of serum 25-hydroxyvitamin D concentrations with cardiovascular and all-cause mortality in individuals with osteoarthritis: results from the NHANES database prospective cohort study. BMC Med. (2022) 20:308. doi: 10.1186/s12916-022-02510-1, PMID: 36127705 PMC9490951

[3] Mendy A Park J Vieira ER. Osteoarthritis and risk of mortality in the USA: a population-based cohort study. Int J Epidemiol. (2018) 47:1821–9. doi: 10.1093/ije/dyy187, PMID: 30169829 PMC7263761

[4] Caughey GE Vitry AI Gilbert AL Roughead EE. Prevalence of comorbidity of chronic diseases in Australia. BMC Public Health. (2008) 8:221. doi: 10.1186/1471-2458-8-221, PMID: 18582390 PMC2474682

[5] Gottlieb DJ Punjabi NM. Diagnosis and Management of Obstructive Sleep Apnea: a review. JAMA. (2020) 323:1389–400. doi: 10.1001/jama.2020.3514 32286648

[6] Mander BA Winer JR Walker MP. Sleep and human aging. Neuron. (2017) 94:19–36. doi: 10.1016/j.neuron.2017.02.004, PMID: 28384471 PMC5810920

[7] Peppard PE Young T Barnet JH Palta M Hagen EW Hla KM. Increased prevalence of sleep-disordered breathing in adults. Am J Epidemiol. (2013) 177:1006–14. doi: 10.1093/aje/kws342, PMID: 23589584 PMC3639722

[8] Mei X Zhao Z Qiu Z Wang J Yu H Zheng C. Association of sleep disorders with clinical symptoms and age in Chinese older adult patients with and without cognitive decline. Front Aging Neurosci. (2023) 15:1189837. doi: 10.3389/fnagi.2023.1189837, PMID: 37621985 PMC10445039

[9] Safiri S Kolahi AA Smith E Hill C Bettampadi D Mansournia MA. Global, regional and national burden of osteoarthritis 1990-2017: a systematic analysis of the global burden of disease study 2017. Ann Rheum Dis. (2020) 79:819–28. doi: 10.1136/annrheumdis-2019-216515, PMID: 32398285

[10] Skou ST Mair FS Fortin M Guthrie B Nunes BP Miranda JJ. Multimorbidity. Nat Rev Dis Primers. (2022) 8:48. doi: 10.1038/s41572-022-00376-4 35835758 PMC7613517

[11] Barnett K Mercer SW Norbury M Watt G Wyke S Guthrie B. Epidemiology of multimorbidity and implications for health care, research, and medical education: a cross-sectional study. Lancet. (2012) 380:37–43. doi: 10.1016/S0140-6736(12)60240-2, PMID: 22579043

[12] March LM Schwarz JM Carfrae BH Bagge E. Clinical validation of self-reported osteoarthritis. Osteoarthr Cartil. (1998) 6:87–93. doi: 10.1053/joca.1997.0098 9692063

[13] Maislin G Pack AI Kribbs NB Smith PL Schwartz AR Kline LR. A survey screen for prediction of apnea. Sleep. (1995) 18:158–66. doi: 10.1093/sleep/18.3.158 7610311

[14] Yang H Watach A Varrasse M King TS Sawyer AM. Clinical trial enrollment enrichment in resource-constrained research environments: multivariable apnea prediction (MAP) index in SCIP-PA trial. J Clin Sleep Med. (2018) 14:173–81. doi: 10.5664/jcsm.6926, PMID: 29246264 PMC5786835

[15] Zhang Q Zhang Q Li X Du G Feng X Ding R. Association of obstructive sleep apnea symptoms with all-cause mortality and cause-specific mortality in adults with or without diabetes: a cohort study based on the NHANES. J Diabetes. (2024) 16:e13538. doi: 10.1111/1753-0407.13538, PMID: 38599827 PMC11006614

[16] Liu Z Kuo PL Horvath S Crimmins E Ferrucci L Levine M. A new aging measure captures morbidity and mortality risk across diverse subpopulations from NHANES IV: a cohort study. PLoS Med. (2018) 15:e1002718. doi: 10.1371/journal.pmed.1002718, PMID: 30596641 PMC6312200

[17] Kwon D Belsky DW. A toolkit for quantification of biological age from blood chemistry and organ function test data: BioAge. Geroscience. (2021) 43:2795–808. doi: 10.1007/s11357-021-00480-5, PMID: 34725754 PMC8602613

[18] Chen L Zhao Y Liu F Chen H Tan T Yao P. Biological aging mediates the associations between urinary metals and osteoarthritis among U.S. adults. BMC Med. (2022) 20:207. doi: 10.1186/s12916-022-02403-3 35710548 PMC9205020

[19] Gaspar LS Álvaro AR Moita J Cavadas C. Obstructive sleep apnea and hallmarks of aging. Trends Mol Med. (2017) 23:675–92. doi: 10.1016/j.molmed.2017.06.006 28739207

[20] Lévy P Kohler M McNicholas WT Barbé F McEvoy RD Somers VK. Obstructive sleep apnoea syndrome. Nat Rev Dis Primers. (2015) 1:15015. doi: 10.1038/nrdp.2015.15 27188535

[21] Chen WJ Liaw SF Lin CC Chiu CH Lin MW Chang FT. Effect of nasal CPAP on SIRT1 and endothelial function in obstructive sleep apnea syndrome. Lung. (2015) 193:1037–45. doi: 10.1007/s00408-015-9790-y 26345325

[22] Pinilla L Santamaria-Martos F Benítez ID Zapater A Targa A Mediano O. Association of Obstructive Sleep Apnea with the aging process. Ann Am Thorac Soc. (2021) 18:1540–7. doi: 10.1513/AnnalsATS.202007-771OC 33662230

[23] Nadeem R Molnar J Madbouly EM Nida M Aggarwal S Sajid H. Serum inflammatory markers in obstructive sleep apnea: a meta-analysis. J Clin Sleep Med. (2013) 9:1003–12. doi: 10.5664/jcsm.3070, PMID: 24127144 PMC3778171

[24] Li Y Wang Y. Obstructive sleep apnea-hypopnea syndrome as a novel potential risk for aging. Aging Dis. (2021) 12:586–96. doi: 10.14336/AD.2020.0723, PMID: 33815884 PMC7990365

[25] Ching K Houard X Berenbaum F Wen C. Hypertension meets osteoarthritis - revisiting the vascular aetiology hypothesis. Nat Rev Rheumatol. (2021) 17:533–49. doi: 10.1038/s41584-021-00650-x 34316066

[26] Barbour KE Lui LY Nevitt MC Murphy LB Helmick CG Theis KA. Hip osteoarthritis and the risk of all-cause and disease-specific mortality in older women: a population-based cohort study. Arthritis Rheumatol. (2015) 67:1798–805. doi: 10.1002/art.39113, PMID: 25778744 PMC4521765

[27] Wei G Lu K Umar M Zhu Z Lu WW Speakman JR. Risk of metabolic abnormalities in osteoarthritis: a new perspective to understand its pathological mechanisms. Bone Res. (2023) 11:63. doi: 10.1038/s41413-023-00301-9, PMID: 38052778 PMC10698167

[28] Silva A Mello MT Serrão PR Luz RP Ruiz F Bittencourt LR. Influence of obstructive sleep apnea in the functional aspects of patients with osteoarthritis. J Clin Sleep Med. (2018) 14:265–70. doi: 10.5664/jcsm.6950, PMID: 29351822 PMC5786847

[29] Carroll JE Irwin MR Seeman TE Diez-Roux AV Prather AA Olmstead R. Obstructive sleep apnea, nighttime arousals, and leukocyte telomere length: the multi-ethnic study of atherosclerosis. Sleep. (2019) 42:zsz089. doi: 10.1093/sleep/zsz089, PMID: 30994174 PMC6612669

[30] Vicente E Marin JM Carrizo SJ Osuna CS González R Marin-Oto M. Upper airway and systemic inflammation in obstructive sleep apnoea. Eur Respir J. (2016) 48:1108–17. doi: 10.1183/13993003.00234-2016 27587551

[31] Loeser RF Collins JA Diekman BO. Ageing and the pathogenesis of osteoarthritis. Nat Rev Rheumatol. (2016) 12:412–20. doi: 10.1038/nrrheum.2016.65, PMID: 27192932 PMC4938009

[32] Chin AF Han J Clement CC Choi Y Zhang H Browne M. Senolytic treatment reduces oxidative protein stress in an aging male murine model of post-traumatic osteoarthritis. Aging Cell. (2023) 22:e13979. doi: 10.1111/acel.13979, PMID: 37749958 PMC10652304

[33] Jeon OH Kim C Laberge RM Demaria M Rathod S Vasserot AP. Local clearance of senescent cells attenuates the development of post-traumatic osteoarthritis and creates a pro-regenerative environment. Nat Med. (2017) 23:775–81. doi: 10.1038/nm.4324, PMID: 28436958 PMC5785239

