# Supplementary material for: Exploring the role of aging in the relationship between obstructive sleep apnea syndrome and osteoarthritis: Insights from NHANES data
Source: Front Med (Lausanne). 2024 Nov 28;11:1486807. doi: 10.3389/fmed.2024.1486807 (PMC11634577; doi:10.3389/fmed.2024.1486807)
Supplement: Supplementary file 1 [file Data_Sheet_1.doc]

STROBE Statement—Checklist of items that should be included in reports of ***cross-sectional studies***

|  | Item No | Recommendation |
| --- | --- | --- |
| **Title and abstract** | 1 | Exploring the role of **aging** in the relationship between obstructive sleep apnea syndrome and osteoarthritis: Insights from NHANES data. |
|  |  | Background: Osteoarthritis (OA) is characterized by high morbidity and disability. Given studies have illuminated OA is correlated with age-related diseases, few research showed the potential relationship between OA and obstructive sleep apnea syndrome (OSAS). OSAS is featured by intermittent hypoxia and hypercapnia, we hypothesized these stressors induced ageing and increased OA prevalence.  Methods: The study included 10641 participants from NHANES dataset in 2005-2008 and 2015-2018 years. The correlation between OSAS and OA was analyzed by multivariable logistic regression, calculated aging-related biomarkers and explored aging role by mediation analysis.  Results: OSAS elevated the risk of OA (for quartile 4 vs. quartile 1, OR 2.31, 95%CI 1.34 to 3.99; P for trend = 0.004) after adjusting covariates; in 20-59 and >60 years subgroup with OSAS patients had similarly trend (for quartile 4 vs. quartile 1, OR 5.69, 95%CI 2.75 to 11.8; P for trend < 0.001; OR 2.42, 95%CI 1.23 to 4.76; P for trend = 0.004, respectively). Further mediation analysis revealed that aging acted as a mediator between OA and OSAS (Biological age and phenotypic age mediation proportion were 13.82% and 52.94%, P < 0.001).  Conclusion: These findings suggested people who had OSAS may boost the prevalence of OA and aging was also involved. |
| Introduction | | |
| Background/rationale | 2 | Osteoarthritis (OA) is a degenerative disease caused by joint cartilage erosion, which results in pain and disability. Estimates suggested that 250 million people had suffered from this disease[1]. Previous investigations have elucidated that elderly individuals diagnosed with arthritis are prone to chronic comorbidities, featuring cardiovascular disorders and hypertension[2-4].  Obstructive sleep apnea syndrome (OSAS) attributed to obesity and airway collapse largely, triggers a cascade of pathophysiological processes including intermittent hypoxia and hypercapnia. These processes subsequently result in hypertension, metabolic disorders, inflammation, which share many similarities with chronic diseases [5].  The incidence of OA and OSA increased with age[6, 7]. As people age, there is a growing trend towards the coexistence of multiple diseases, making the treatment process more challenging [8, 9]. Although many studies have reported that OA is associated with chronic disease, few research has showed the association between OA and OSA in large number of participants, the role of aging in these two diseases is yet unknown. |
| Objectives | 3 | we attempted to use the National Health and Nutrition Examination Survey (NHANES, 2005-2008 and 2015-2018) database to explore the risk of OA and OSA and the role of aging in this process. By managing OSA and delaying aging, we try to relieve pain and improve the quality of life for elderly patients with OA. |
| Methods | | |
| Study design | 4 | Nationwide cross-sectional study |
| Setting | 5 | We selected the United States 10641 participants in the NHANES cycles of 2005-2008 and 2015-2018. |
| Participants | 6 | Selected participants from NHANES meeting the follow criteria:  1). aged >20 years old;  2). including OSA and OA questionnaires information;  3). including age, sex, race, education, family poverty income ratio (PIR), smoking, drinking, BMI and blood pressure data;  4). including self-reported medical history, medication use information. |
| Variables | 7 | Outcomes: OA (self-reported arthritis and the type of arthritis was osteoarthritis)  Exposures: OSA (based on one of three symptoms: (1) snoring 3 or more nights per week; (2) snorting or stopping breathing 3 or more nights per week; (3) feeling overly sleepy during the day 16-30 times a month despite sleeping around 7 or more hour per night on weekdays or work nights)  Effect modifiers: aging |
| Data sources/ measurement | 8* | NHANES questionnaires:  OA group (self-reported osteoarthritis); non-OA group (other types of arthritis or reporting an absence of arthritis)  OSAS index and OSAS.MAP10: We referred to the previous study of Maislin G et al to calculate MAP index and OSAS.MAP10 based on one of three symptoms: (1) snoring 3 or more nights per week; (2) snorting or stopping breathing 3 or more nights per week; (3) feeling overly sleepy during the day 16-30 times a month despite sleeping around 7 or more hour per night on weekdays or work nights^[10]^. They evaluated the risk of OSAS through a multivariable apnea prediction (MAP) index (0 - 1.0), based on the first two questions. According to participant snoring frequency, these items were scored 0, 2, 3, 4 (never, rarely 1-2 nights per week, occasionally 3-4 nights a week, frequently 5 or more nights a week). The MAP index formula is: MAP index =e^x^ / (1 + e^x^), where x = - 8.16 + 1.299 × Index 1 + 0.163 × body mass index(c) - 0.028 × Index 1× Index 1 + 0.032 × Age + 1.278 × Sex, and where sex = 1 if male and 0 if female, Index 1 is the mean score of the two self-reported items^[11]^. For better explanation in this study, multiply the MAP index value by 10 and define it as OSAS.MAP10.  biological aging: Previous studies have found that KDM biological age and phenotypic age can better predict individual aging levels through a series of algorithms^[12]^. We first extracted individual KDM biological age biomarkers and chronological age. Then, we calculated KDM biological age and phenotypic age using the BioAge R package, which was downloaded at GitHub https://github.com/dayoonkwon/BioAge/)^[13]^. The NHANES III data were used to calculate KDM biological age, and then used our data to fit the aging model ^[13]^. Likewise, we extracted individual biomarkers, using the BioAge R package calculated phenotypic age (supplementary materials for details). |
| Bias | 9 | There was recall bias because OA questionnaires is self-reported, data were selected from all cycles containing information on both OSA and OA, and follow-up studies may be conducted to explore whether the risk of OA in OSA patients is reduced with OSA management. We set three models to control covariates. Model 1: no covariates were adjusted. Model 2: adjusted for age, sex, race, education level, PIR, BMI, smoking status, alcohol consumption. Model 3: adjusted for age, sex, race, education level, PIR, BMI, smoking status, alcohol consumption, CVD (coronary heart disease, stroke, heart attack, chronic heart failure, angina pectoris), hypertension, diabetes, COPD; obstructive sleep apnea syndrome. |
| Study size | 10 | NHANES selected representative samples by stratified sampling, from which we selected 10641 subjects who met the inclusion criteria of this study. |
| Quantitative variables | 11 | We group OSAS.MAP10 into quartiles to explore the impact of OSAS severity on OA. |
| Statistical methods | 12 | This study used R studio (version 4.4.0) for statistical analysis of weighted data. We grouped the subjects according to their osteoarthritis status, using the Wilcoxon rank-sum test and the Chi-squared test for demographic analysis. We used quartiles to convert OSAS.MAP10 continuous-type variables into categorical variables. Multivariable logistic regression was used to the estimate odds ratio (OR) and the corresponding 95% confidence interval (CI) for the associations of OSAS.MAP10 and OA risk after adjusted covariates. Then, we explored the mediating effect of aging on OSAS.MAP10 and OA are done using the mediation R package. Mediation analyses used the quasi-Bayesian Monte Carlo method with 1000 simulations based on normal approximation. The direct effect (DE) represented OSA effects on OA directly without aging mediation. The indirect effect (IE) indicated that OSA acted on OA through aging mediation. The mediation proportion was calculated using IE and divided by TE (total effect). Excluded missing the data of OSA, OA, aging biomarkers. |
| Results | | |
| Participants | 13* | (a) 39722 Participants were selected in 2005-2008 and 2015-2018 cycles, in these cycles contained OSAS and OA related questionnaires. We selected 21861 participants aged >20 years. |
|  |  | (b) Because of in this study variables were OSAS and OA, we removed 1316 participants who missing these two variables, and excluded 9904 participants missing data of covariables. |
|  |  | (c) 10641 Final participants were obtained for the follow-up study (Figure 1 flow chart for details). |
| Descriptive data | 14* | In this study, we screened 1027 patients over the age of 20 with osteoarthritis (mean age: 60.47 ± 12.65; 37.01% males). Of them, the OSAS.MAP10 score of 4.82 ± 2.54. Table 1 showed demographic characteristics according to OA status. Patients characterized by older, female, high-income, obese, smoking, and drinking were more likely to have osteoarthritis (*P* < 0.001). |
| Outcome data | 15* | Dependent variable: OA patients, who self-reported had osteoarthritis. |
| Main results | 16 | Associations between OSAS and OA risk: The highest OSAS.MAP10 quantile of (OR 3.48, 95%CI 2.60 to 4.65) increased the risk of OA compared to quantile 1 (all *P* for trend < 0.005) in unadjusted model 1. The trend did not change in model 2, which adjusted for age, sex, race, education level, PIR, BMI, smoking status, and alcohol consumption. Model 3 shared similarly trend, which adjusted for all variables in model 2 and other risk factors for CVD (coronary heart disease, stroke, heart attack, chronic heart failure, angina pectoris), hypertension, diabetes, COPD. |
| Other analyses | 17 | The associations between OSAS and OA risk in age and sex subgroup: Table 3 showed the associations between OSAS and OA risk in different age and sex subgroups. We found the highest quartile of OSAS.MAP10 (OR 3.08, 95%CI 2.04 to 4.64, P < 0.001) in 20-59 years subgroup increased OA risk after adjusted in model 1, compared to quartile 1, model 2 (OR 8.18, 95%CI 4.24 to 15.8, P < 0.001) and model 3 (OR 5.69, 95%CI 2.75 to 11.8, P < 0.001) had the same trend. However, OSAS.MAP10 in the 60-year-old subgroup did not share the same trend (OR 1.01, 95%CI 0.58 to 1.77, P = 0.664) in model 1. Adjusting for the covariates, OSAS.MAP10 restored the trend in model 2 (OR 2.29, 95% CI 1.19 to 4.38, P = 0.006) and model 3 (OR 2.42, 95% CI 1.23 to 4.76, P = 0.004). Table 4 showed the highest quartile of OSAS.MAP10 in the male subgroup increased OA risk in three models (P < 0.005). However, the quartile 4 of OSAS.MAP10 in the female subgroup increased OA risk in model 1 (OR 6.24, 95%CI 4.26 to 9.14, P < 0.001) and model 2 (OR 2.32, 95%CI 1.25 to 4.31, P = 0.043), there was no significant difference in model 3 (OR 2.16, 95%CI 1.14 to 4.07, P = 0.096).  Aging-mediated effects on the association of OSA with OA risk: Furthermore, we used R package to explore the aging mediating role between OSAS.MAP10 and OA in figure 2. Biological age and phenotypic age mediated the associations of OSAS.MAP10 with OA risk with 13.82% and 52.94% proportion of mediation, respectively (all P < 0.001). |
| Discussion | | |
| Key results | 18 | Our results found that OSAS may increase the prevalence of OA. explored the aging mediating effects on these two diseases. |
| Limitations | 19 | Firstly, we cannot determine causality between OSAS and OA because of our study was a cross-sectional design. Secondly, information on OA and OSAS was self-reported, which caused recall bias and decreased the credibility of the results. Thirdly, although we adjusted for the survey cycle, there are four survey cycles, which were limited to only in these cycles that had self-reported both OA and OSAS questionnaires. |
| Interpretation | 20 | Therefore, follow-up studies are needed to validate, analyse, and support our findings. |
| Generalisability | 21 | This study may provide a new perspective for the management of multiple diseases in the elderly. Managing OSA may delaying aging, relieve pain and improve the quality of life in elderly patients with OA. |
| Other information | | |
| Funding | 22 | This study was supported by the Research Initiation Fundings of Anhui Provincial Hospital (Grant No. RC2023036). |

*Give information separately for exposed and unexposed groups.

**Note:** An Explanation and Elaboration article discusses each checklist item and gives methodological background and published examples of transparent reporting. The STROBE checklist is best used in conjunction with this article (freely available on the Web sites of PLoS Medicine at http://www.plosmedicine.org/, Annals of Internal Medicine at http://www.annals.org/, and Epidemiology at http://www.epidem.com/). Information on the STROBE Initiative is available at www.strobe-statement.org.

[1] Hunter D J, Bierma-Zeinstra S. Osteoarthritis [J]. Lancet, 2019, 393(10182): 1745-59.

[2] Xiao Q, Cai B, Yin A, Huo H, Lan K, Zhou G, et al. L-shaped association of serum 25-hydroxyvitamin D concentrations with cardiovascular and all-cause mortality in individuals with osteoarthritis: results from the NHANES database prospective cohort study [J]. BMC Med, 2022, 20(1): 308.

[3] Mendy A, Park J, Vieira E R. Osteoarthritis and risk of mortality in the USA: a population-based cohort study [J]. Int J Epidemiol, 2018, 47(6): 1821-9.

[4] Caughey G E, Vitry A I, Gilbert A L, Roughead E E. Prevalence of comorbidity of chronic diseases in Australia [J]. BMC Public Health, 2008, 8(221.

[5] Gottlieb D J, Punjabi N M. Diagnosis and Management of Obstructive Sleep Apnea: A Review [J]. Jama, 2020, 323(14): 1389-400.

[6] Peppard P E, Young T, Barnet J H, Palta M, Hagen E W, Hla K M. Increased prevalence of sleep-disordered breathing in adults [J]. Am J Epidemiol, 2013, 177(9): 1006-14.

[7] Safiri S, Kolahi A A, Smith E, Hill C, Bettampadi D, Mansournia M A, et al. Global, regional and national burden of osteoarthritis 1990-2017: a systematic analysis of the Global Burden of Disease Study 2017 [J]. Ann Rheum Dis, 2020, 79(6): 819-28.

[8] Skou S T, Mair F S, Fortin M, Guthrie B, Nunes B P, Miranda J J, et al. Multimorbidity [J]. Nat Rev Dis Primers, 2022, 8(1): 48.

[9] Barnett K, Mercer S W, Norbury M, Watt G, Wyke S, Guthrie B. Epidemiology of multimorbidity and implications for health care, research, and medical education: a cross-sectional study [J]. Lancet, 2012, 380(9836): 37-43.

[10] Maislin G, Pack A I, Kribbs N B, Smith P L, Schwartz A R, Kline L R, et al. A survey screen for prediction of apnea [J]. Sleep, 1995, 18(3): 158-66.

[11] Yang H, Watach A, Varrasse M, King T S, Sawyer A M. Clinical Trial Enrollment Enrichment in Resource-Constrained Research Environments: Multivariable Apnea Prediction (MAP) Index in SCIP-PA Trial [J]. J Clin Sleep Med, 2018, 14(2): 173-81.

[12] Liu Z, Kuo P L, Horvath S, Crimmins E, Ferrucci L, Levine M. A new aging measure captures morbidity and mortality risk across diverse subpopulations from NHANES IV: A cohort study [J]. PLoS Med, 2018, 15(12): e1002718.

[13] Kwon D, Belsky D W. A toolkit for quantification of biological age from blood chemistry and organ function test data: BioAge [J]. Geroscience, 2021, 43(6): 2795-808.
