# Supplementary material for: Exploring the role of aging in the relationship between obstructive sleep apnea syndrome and osteoarthritis: Insights from NHANES data
Source: Front Med (Lausanne). 2024 Nov 28;11:1486807. doi: 10.3389/fmed.2024.1486807 (PMC11634577; doi:10.3389/fmed.2024.1486807)
Supplement: Supplementary file 2 [file Data_Sheet_2.docx]

Supplementary Material

Xin Luo, Minghong Chen, Jinghong Xu. Exploring the role of **aging** in the relationship between obstructive sleep apnea syndrome and osteoarthritis: Insights from NHANES data.

# Methods

We downloaded and cleansed data of interest, including questionnaires, physical examinations, and laboratory tests in 2005-2008 and 2015-2018 cycles.

## Calculated MAP index and OSAS.MAP10

We referd to the previous study of Maislin G et al to calculate MAP index and OSAS.MAP10 based on one of three symptoms: (1) snoring 3 or more nights per week; (2) snorting or stopping breathing 3 or more nights per week; (3) feeling overly sleepy during the day 16-30 times a month despite sleeping around 7 or more hour per night on weekdays or work nights^[1]^. They evaluated the risk of OSAS through a multivariable apnea prediction (MAP) index (0 - 1.0), based on the first two questions. According to participant snoring frequency, these items were scored 0, 2, 3, 4 (never, rarely 1-2 nights per week, occasionally 3-4 nights a week, frequently 5 or more nights a week). The MAP index formula is: MAP index =e^x^ / (1 + e^x^), where x = - 8.16 + 1.299 × Index 1 + 0.163 × body mass index(c) - 0.028 × Index 1× Index 1 + 0.032 × Age + 1.278 × Sex, and where sex = 1 if male and 0 if female, Index 1 is the mean score of the two self-reported items^[2]^. For better explanation in this study, multiply the MAP index value by 10 and define it as OSAS.MAP10.

## Calculated biological aging markers

Previous studies have found that KDM biological age and phenotypic age can better predict individual aging levels through a series of algorithms^[3]^.

1. The KDM BA algorithm is derived from a series of regressions of individual biomarkers. We obtained biomarkers, including C-reactive protein, serum creatinine, glycosylated hemoglobin, serum albumin, serum total cholesterol, serum urea nitrogen, serum alkaline phosphatase, and systolic blood pressure, and chronological age.
2. Then, we calculated KDM biological age using the BioAge R package, which was downloaded at GitHub (https://github.com/dayoonkwon/BioAge/)^[4]^. The NHANES III data were used to calculate KDM biological age, and then used our data to fit the aging model ^[4]^.

$$\text{KDM-BA=}\frac{\sum_{\text{i=1}}^{\text{n}} \text{(x}_{\text{i}}\text{-}\text{q}_{\text{i}}\text{)}\frac{\text{k}_{\text{i}}}{\text{s}_{\text{i}}^{\text{2}}}\text{+}\frac{\text{CA}}{\text{s}_{\text{BA}}^{\text{2}}}}{\sum_{\text{i=1}}^{\text{n}} {\text{(}\frac{\text{k}_{\text{i}}}{\text{s}_{\text{i}}}\text{)}}^{\text{2}}\text{+}\frac{\text{1}}{\text{s}_{\text{BA}}^{\text{2}}}}$$

where x is the value of biomarker i measured for an individual. For each biomarker i, the parameters k, q, and s are estimated from a regression of chronological age on the biomarker in the reference sample. k, q, and s are the regression intercept, slope, and root mean squared error, respectively. sBA is a scaling factor equal to the square root of the variance in chronological age explained by the biomarker set in the reference sample. CA is chronological age.

3. Likewise, we extracted individual biomarkers, using the BioAge R package calculated phenotypic age. Phenotypic age was calculated based on chronological age using 9 multi-system clinal chemistry biomarker (albumin, creatinine, glucose, C-reactive protein, lymphocyte percent, mean cell volume, red blood cell distribution width, alkaline phosphatase, and white blood cell count) ^[3]^. The formula for PhenoAge computation is as follows:

$$\text{PhenoAge}\text{ = 141.50225 + }\frac{\ln\left[ \text{- 0.00553 × }\ln\left( \text{1 - mortality risk} \right) \right]}{\text{0.090165}}$$

where xb = - 19.907 - 0.0336 × Albumin + 0.0095 × Creatinine + 0.1953 × Glucose + 0.0954 × LnCRP - 0.0120 × Lymphocyte percent + 0.0268 × Mean Cell Volume + 0.3306 × Red Cell Distribution Width + 0.00188 × Alkaline Phosphatase + 0.0554 × White Blood Cell Count + 0.0804 × Chronological Age.

[1] Maislin G, Pack A I, Kribbs N B, Smith P L, Schwartz A R, Kline L R, et al. A survey screen for prediction of apnea [J]. Sleep, 1995, 18(3): 158-66.

[2] Yang H, Watach A, Varrasse M, King T S, Sawyer A M. Clinical Trial Enrollment Enrichment in Resource-Constrained Research Environments: Multivariable Apnea Prediction (MAP) Index in SCIP-PA Trial [J]. J Clin Sleep Med, 2018, 14(2): 173-81.

[3] Liu Z, Kuo P L, Horvath S, Crimmins E, Ferrucci L, Levine M. A new aging measure captures morbidity and mortality risk across diverse subpopulations from NHANES IV: A cohort study [J]. PLoS Med, 2018, 15(12): e1002718.

[4] Kwon D, Belsky D W. A toolkit for quantification of biological age from blood chemistry and organ function test data: BioAge [J]. Geroscience, 2021, 43(6): 2795-808.

**
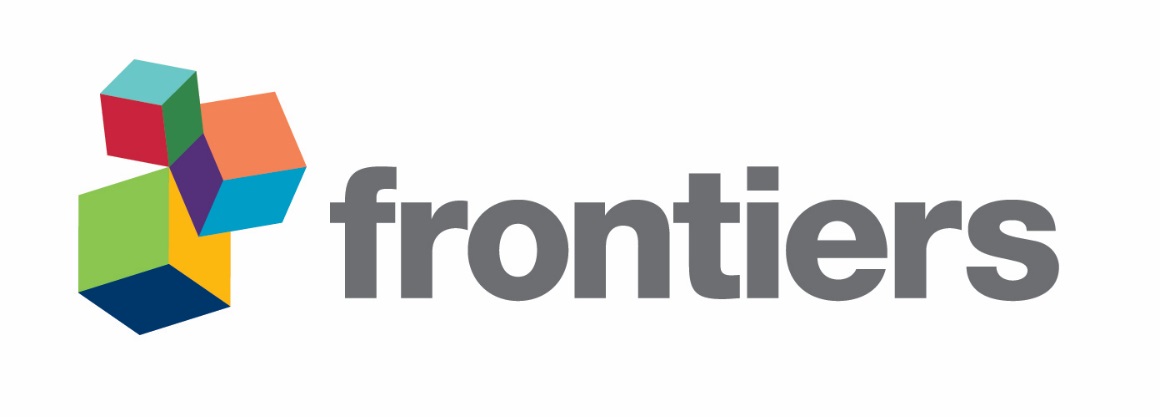
**
